# Birth outcomes in women who have taken adalimumab in pregnancy: A prospective cohort study

**DOI:** 10.1371/journal.pone.0223603

**Published:** 2019-10-18

**Authors:** Christina D. Chambers, Diana L. Johnson, Ronghui Xu, Yunjun Luo, Janina Lopez-Jimenez, Margaret P. Adam, Stephen R. Braddock, Luther K. Robinson, Keith Vaux, Kenneth Lyons Jones, the OTIS Collaborative Research Group

**Affiliations:** 1 Department of Pediatrics, University of California San Diego, La Jolla, CA, United States of America; 2 Department of Family Medicine and Public Health, University of California San Diego, La Jolla, CA, United States of America; 3 Department of Mathematics, University of California San Diego, La Jolla, CA, United States of America; 4 Department of Pediatrics, University of Washington, Seattle, WA, United States of America; 5 Deparment of Pediatrics, Saint Louis University, St. Louis, MO, United States of America; 6 Department of Pediatrics, State University of New York at Buffalo, Buffalo, NY, United States of America; Brigham and Women's Hospital, UNITED STATES

## Abstract

**Background:**

Information is needed on the safety of adalimumab when used in pregnancy for the treatment of certain autoimmune diseases.

**Methods and findings:**

Between 2004 and 2016, the Organization of Teratology Information Specialists Research Center at the University of California San Diego conducted a prospective controlled observational cohort study in 602 pregnant women who had or had not taken adalimumab. Women in the adalimumab-exposed cohort had received at least one dose of the drug in the first trimester for the treatment of rheumatoid arthritis or Crohn’s Disease (N = 257). Women in the disease comparison cohort had not used adalimumab in pregnancy (N = 120). Women in the healthy comparison cohort had no rheumatic or inflammatory bowel diseases (N = 225). Women and their infants were followed to one year postpartum with maternal interviews, medical records abstraction, and physical examinations. Study outcomes were major structural birth defects, minor defects, spontaneous abortion, preterm delivery, pre and post-natal growth deficiency, serious or opportunistic infections and malignancies. 42/602 (7.0%) of pregnancies were lost-to-follow-up. 22/221 (10.0%) in the adalimumab-exposed cohort had a live born infant with a major birth defect compared to 8/106 (7.5%) in the diseased unexposed cohort (adjusted odds ratio 1.10, 95% confidence interval [CI] 0.45 to 2.73). Women in the adalimumab-exposed cohort were more likely to deliver preterm compared to the healthy cohort (adjusted hazard ratio [aHR] 2.59, 95% CI 1.22 to 5.50), but not compared to the diseased unexposed cohort (aHR 0.82, 95% CI 0.66 to 7.20). No significant increased risks were noted with adalimumab exposure for any other study outcomes.

**Conclusions:**

Adalimumab exposure in pregnancy compared to diseased unexposed pregnancies was not associated with an increased risk for any of the adverse outcomes examined. Women with rheumatoid arthritis or Crohn’s Disease were at increased risk of preterm delivery, irrespective of adalimumab exposure.

## Introduction

Anti-tumor necrosis factor alpha (anti-TNF-α) therapies have been available for the treatment of various chronic inflammatory diseases for over 20 years. Many of these diseases are prevalent in women of child-bearing age. For this reason, evaluation of the safety of anti-TNF-α therapies used in pregnancy is needed. This includes information on drug-specific risks of major birth defects, pregnancy loss, preterm delivery, and pre-and postnatal growth deficiency. In addition, due to the immunosuppressive action of TNF inhibitors, it is also relevant to examine risks for serious or opportunistic infections and malignancies in infants who have had prenatal exposure to an anti-TNF-α medication.

Adalimumab is an anti-TNF-α medication first approved in the U.S. in 2002 for the treatment of rheumatoid arthritis and subsequently indicated for psoriatic arthritis, ankylosing spondylitis, Crohn's Disease (adult and pediatric), ulcerative colitis, plaque psoriasis, hidradenitis suppurativa (adult and adolescent), non-infectious uveitis (adult and pediatric), and juvenile idiopathic arthritis. Adalimumab is a fully humanized monoclonal antibody with a high molecular weight and is expected to require active transport in order to cross the human placenta. For this reason, it is thought that potential exposure of the embryo via placental transfer is limited earlier in pregnancy, while transfer to the fetus later in pregnancy has been documented [1].

There are limited data regarding the fetal safety of adalimumab when used in human pregnancy. Two published studies are notable. One study conducted in nine countries compared birth outcomes in 495 women exposed to any anti-TNF-α therapy in the first trimester to outcomes in 1,532 women without chronic inflammatory diseases who had consulted with a European or Australian Teratology Information Service between 1998 and 2013. The investigators found a 2.2-fold increased risk (95% Confidence Interval [CI] 1.0 to 4.8) for all major birth defects combined, and an increased risk of preterm birth and low birth weight in the exposed compared to unexposed. However, there was no specific pattern of major birth defects noted in exposed infants nor any differential risk for major birth defects in the subset of 177 women in the sample who were exposed to adalimumab [2]. Furthermore, the comparison group in this study was not matched on maternal disease.

A second study combined data from Danish and Swedish population health registers for 1,272,424 pregnancies ending in live birth between 2004–2012. The risk of major birth defects overall was compared between 683 women treated with any anti-TNF-α medication for chronic inflammatory disease and 21,549 women with chronic inflammatory disease not treated with any anti-TNF-α therapy. The adjusted odds ratio (OR) for major birth defects was 1.32 (95% CI 0.83 to 1.82) for exposed vs. disease-matched unexposed. There was no evidence of a specific pattern of major birth defects with exposure to any anti-TNF-α medication. The adjusted OR for major birth defects in the subset of 161 adalimumab-exposed pregnancies compared to women with chronic inflammatory disease and no anti-TNF-α treatment was 1.22 (95% CI 0.58 to 2.27) [3].

Taken together, these studies suggest that the underlying maternal inflammatory diseases may play a role in birth outcomes in women treated with anti-TNF-α medication in pregnancy. However, to our knowledge no study to date has comprehensively examined the range of pregnancy outcomes among pregnancies specifically exposed to adalimumab compared to disease-matched and healthy women without the same chronic inflammatory diseases. The purpose of this study was to evaluate the birth prevalence and patterns of major and minor structural birth defects, spontaneous abortion, preterm birth, pre- and postnatal growth deficiency, and risks for serious or opportunistic infections or malignancies in infants born to mothers treated with adalimumab during part or all of pregnancy.

## Methods

### Design and setting

MotherToBaby pregnancy studies conducted by the Organization of Teratology Information Specialists (OTIS) are prospective pregnancy cohort studies involving study participants across the U.S. and Canada. The methods of MotherToBaby OTIS cohort studies have been described previously [4–6].

In brief, MotherToBaby services, located in academic institutions or hospitals throughout the U.S. and Canada, provide counseling to women and their health care providers who contact the services with questions about the risks of exposures in pregnancy. Pregnant women who meet the criteria for a study are referred to the OTIS Research Center at the University of California San Diego where they are screened, consented to participate and where all subsequent data collection takes place. Additional methods of recruitment are also employed, including physician referrals and direct-to-consumer marketing through social media and the MotherToBaby website. In all cases, the pregnant woman is the individual who provides consent to participate in the study and she is the primary source of medication exposure data.

### Patient and public involvement

MotherToBaby/OTIS pregnancy studies select topics for research in part based on input from patients and the public. This includes assessment of public and patient interest based on the frequency of queries received by MotherToBaby/OTIS services about a specific medication, the specific concerns raised, and whether or not there is a lack of adequate human pregnancy data for a given exposure. Women who enroll in MotherToBaby/OTIS pregnancy studies are asked to refer others who may have interest in the study. Participants in MotherToBaby/OTIS pregnancy studies are formally surveyed about their experience and satisfaction with the study procedures, and asked about reasons for decline to participate or withdrawals. These factors are considered in designing or amending the study procedures. Finally, mothers receive feedback on the results of the study-related physical examinations for their infants, and all participants are provided overall study results.

### Participants

A Pregnancy Registry study for adalimumab was initiated in January, 2004, and enrollment continued until August, 2016. Women with a diagnosis of rheumatoid arthritis or Crohn’s Disease and prenatal exposure to adalimumab were prospectively recruited into an exposed cohort. Women with the same diagnoses but no treatment with adalimumab at any time in pregnancy were recruited into a disease comparison cohort. An additional cohort of healthy comparison women without rheumatic or inflammatory bowel diseases was also recruited and followed in the same manner.

Women met the inclusion criteria for all three cohorts if they enrolled in the study prior to 20 weeks’ gestation, did not have prenatal diagnosis prior to enrollment with this pregnancy of a fetus with a major structural birth defect, and had not enrolled in the study with a previous pregnancy. Inclusion criteria for the adalimumab-exposed group required receipt of any dose of adalimumab at any time in pregnancy from the date of conception (DOC) to the end of the first trimester defined as 11 weeks after the estimated date of conception, with or without continued use of the medication through the remainder of gestation. Women met the inclusion criteria for the disease comparison group if they had a diagnosis of rheumatoid arthritis or Crohn’s Disease, but were not treated with adalimumab at any time in pregnancy, although they could have been treated with another anti-TNF agent such as etanercept. Women enrolled in the healthy cohort did not have a rheumatic disease or an inflammatory bowel disease, no treatment with a monoclonal antibody medication in pregnancy and no exposure to a known human teratogen in pregnancy at the time of enrollment. Women in this cohort were primarily recruited from MotherToBaby services from callers who initiated contact with the service about non-teratogenic exposures, e.g., cosmetic products, an antibiotic or over-the-counter cold preparation.

### Maternal interviews

Women in both the exposed and unexposed cohorts completed up to three telephone interviews during pregnancy and one interview at the completion of pregnancy. These were administered by trained study staff in English or Spanish. The first interview obtained information on demographic, pregnancy and family medical history, tobacco, alcohol and caffeine consumption, and recreational drug use, as well as infections, fever, and prenatal tests. Data regarding products, dosages, dates and indications for exposure were collected on all medications (prescription, over-the-counter), vitamins/minerals, herbal products, and vaccines used/administered from the first day of the last menstrual period (LMP) through the date of the interview. Subsequent maternal interviews elicited information on additional exposures or events since the last interview. The Slone Epidemiology Center’s Drug Dictionary was used to code exposures [7].

In the exposed and disease comparison cohorts, women also responded to questionnaires at enrollment and in the third trimester interview about disease activity or symptom control. For women with rheumatoid arthritis, patient-reported measures consisted of the Health Assessment Questionnaire with Disability Index (HAQ-DI) [8], a measure of disease-related pain, and the global impact of the disease. As the study progressed, Crohn’s Disease was added as an indication for the exposed cohort. From that point forward, women with this condition completed the Short Inflammatory Bowel Disease Questionnaire (SIBDQ) [9]. However, women with Crohn’s Disease who met criteria for the cohort, but who had enrolled in the study prior to the addition of Crohn’s did not have the SIBDQ administered.

### Outcomes

Outcomes were collected by maternal interview and by medical records obtained from the obstetrician, rheumatologist or gastroenterologist, pediatrician, and delivery hospital as well as pathology reports if relevant. Data were collected on outcome status of each pregnancy (live birth, stillbirth, spontaneous abortion, and elective termination), gestational age at outcome, mode of delivery, sex and number of infants, birth weight, length, and head circumference and the presence or absence of major birth defects detected up through the first year of life. Maternal report of major birth defects was confirmed by medical record review.

Major structural defects were reviewed by a birth defects specialist who was a co-investigator for this study (KLJ) and final classification was made using the U.S. Centers for Disease Control and Prevention Metropolitan Atlanta Congenital Defects Program coding system [10]. Spontaneous abortion was defined as spontaneous pregnancy loss at <20.0 gestational weeks. Preterm delivery was defined as delivery at <37.0 gestational weeks. Ultrasound dating was used to correct gestational weeks, as necessary for discrepant dates or if the LMP was unknown using a standard algorithm. Small for gestational age infants on weight, length and head circumference was defined as ≤10^th^ centile for sex and gestational age in live born infants using standard U.S. growth charts for full and preterm infants [11–13].

Minor structural defects were evaluated through a study-specific physical examination of live born infants conducted by one of a team of pediatric specialists in dysmorphology/genetics. Examinations were performed most commonly in the participant’s home, and the examiners were blinded to the mother’s/infant’s exposure status at the time of the evaluation. A study-specific checklist of minor structural defects (S1 Table) was used to document the presence or absence of approximately 132 minor features. In addition, infant length and head circumference were measured and infant photographs were taken if the parent provided specific consent.

Live born children were routinely followed for one year post-partum. Medical records from the pediatrician were requested and abstracted for additional evidence of any major structural birth defects, postnatal growth, serious or opportunistic infections, and malignancies. Postnatal growth deficiency was defined as ≤10^th^ centile for weight, length and head circumference for sex and chronological age at about one year using standard U.S. growth charts. Serious or opportunistic infections were defined as infections requiring hospitalization or those from a specific checklist (S2 Table).

Participants provided informed consent. Institutional review board approval for the study was obtained through the University of California San Diego, La Jolla, California. The study protocol was posted to the U.S. Clinicaltrials.gov under registration number NCT01086059. Annual interim, final results and conclusions of the study investigators were reviewed by an independent Scientific Advisory Board with expertise in epidemiology, congenital malformations and maternal autoimmune diseases.

### Statistical methods

The primary statistical comparison was performed between the group of women exposed to adalimumab and the women in the disease comparison cohort, with subsequent comparisons within the two disease strata (rheumatoid arthritis and Crohn’s Disease) for the outcome of major structural birth defects. The unit of analysis for major structural birth defects was the pregnancy, i.e., a singleton or multiple pregnancy that ended in one or more malformed infants was considered one major birth defect outcome. For minor structural birth defects, the analysis was restricted to infants for whom the study-related physical examination was completed. The prevalence of any three or more minor structural birth defects was compared between the adalimumab-exposed infants and the unexposed infants. In addition, among the adalimumab-exposed, clusters of any two or more infants with the exact same three or more minor structural defects were identified and the prevalence of those patterns was compared to the same specific clusters in the unexposed.

Secondary comparisons for all outcomes were performed between the group of women exposed to adalimumab and the healthy cohort.

Exact methods were used to calculate crude relative risks (RRs) and their 95% CIs for the pregnancy outcomes of major birth defects overall, minor structural defects, small for gestational age infants, and infants with serious or opportunistic infections or malignancies. Adjusted RRs and their 95% CIs were estimated when numbers permitted using logistic regression with the adjusted odds ratios (ORs) used as an approximation of the adjusted RRs.

For spontaneous abortion, Kaplan Meier estimates were used to estimate the probability of spontaneous abortion accounting for left truncation due to varying gestational timing of enrollment, and data were censored at 20 weeks’ gestation [14]. Cox proportional hazards modeling was used to estimate hazard ratios (HRs) and their 95% CIs. Similar methods were used for preterm delivery, where data were censored at 37 weeks’ gestation. Twins or higher order multiples were excluded in the analyses of preterm delivery and small for gestational age infants due to the inherent higher risk for these adverse outcomes in multiple gestations.

Control for confounding was conducted where numbers permitted. A minimum of 30 events for a binary outcome was considered the threshold for conducting adjusted analyses. A minimum of 20 events was required for Cox regression as it does not contain an intercept. All relevant covariates for each specific outcome including maternal age (categorical according to standard age groupings), race, ethnicity, socioeconomic status (categorical based on high vs lower Hollingshead categories), year of enrollment (categorical based on distribution of enrollment years), referral source, country of residence, tobacco or alcohol use in pregnancy, pre-pregnancy body mass index (categorical using standard groupings), use of vitamin supplements, pregnancy history, infection, fever, psychiatric conditions, use of corticosteroids (any, average dose across weeks’ taken, number of weeks used), use of other immunosuppressant medications, exposure to known human teratogens (e.g., methotrexate), and comorbid autoimmune diseases were considered as possible confounders. A criterion of ≥10% change in the estimate of the OR or the HR for the outcome under consideration with the addition of each covariate in a model containing exposure to adalimumab (yes/no) was used to select potential confounders for each outcome. If one confounder was identified, direct adjustment was performed. If two or more confounders were identified for a particular outcome, a propensity score approach was used. In this approach, the selected confounders for each analysis were used to build the propensity score model, and the propensity score for each woman was then used as a single adjustment factor in the regression models [15, 16]. Propensity scores were computed using R package ‘twang’. Balance of confounders between the exposed and unexposed groups was assessed by computing standardized mean differences. Adjusted ORs or HRs and their 95% CIs were then computed using logistic or Cox regression adjusted for the logit of the propensity score.

Disease severity/symptom control measures were used to evaluate comparability of the adalimumab-exposed and disease-matched comparison women on disease status. However, it was not possible to collect these measures at baseline (i.e., prior to initiation of any treatment for maternal disease). Therefore, in the final adjusted analyses disease severity measures were not considered as confounders as they could have been influenced by treatment.

Missing values were excluded from each analysis (S4 Table). However, for the outcome of spontaneous abortion, if the exact gestational age at the time of pregnancy loss was missing, multiple imputation was used for the date of the event.

For the primary outcome of major birth defects, sensitivity analyses were conducted in five ways: 1) logistic regression with adjustment for the logit of a propensity score comprised of all considered confounders, 2) inverse probability weighting with the propensity score comprised of only the selected confounders, 3) inverse probability weighting with the propensity score comprised of all considered confounders, 4) restricting the analysis to women who enrolled prior to any prenatal diagnostic test for major birth defects, and 5) excluding women from the disease-matched unexposed group who had exposure to another anti-TNF-α medication in pregnancy.

All analyses were conducted using R open-source statistical software or StatXact (2011).

### Ethical approval

This study was approved by the University of California, San Diego’s institutional review board (approval number 150226). The study was conducted according to the current regulations of the International Conference on Harmonization guidelines and the principles of the Declaration of Helsinki. Informed consent was obtained from participants.

## Results

Between 2004 and 2016, 602 pregnant women enrolled in the study; 257 were exposed to adalimumab in pregnancy, 120 were disease-matched unexposed, and 225 were healthy comparison pregnancies. A total of 42/602 of enrolled women (7.0%) were lost-to-follow-up primarily due to inability to obtain birth outcome information from the participant despite repeated attempts to contact up to one year after the estimated date of delivery. Although all women in the adalimumab-exposed cohort were exposed for some period of time in the first trimester, 65% of the sample used the medication in all three trimesters (Table 1). The distribution of timing of exposure to adalimumab across gestation is shown in S1 Fig.

**Table 1:** Selected maternal characteristics of women exposed during pregnancy to adalimumab and pregnant women not exposed, 2004–2016.

Characteristic	Adalimumab-Exposed N = 257 n (%)	Diseased Unexposed N = 120 n (%)	p-value^a^	Healthy Unexposed N = 225 N (%)	p-value^a^
**Maternal Age—years**					
<25	14 (5.4)	9 (7.5)		18 (8.0)	
25–29	81 (34.5)	28 (23.3)	0.380	54 (24.0)	0.221
30–34	88 (34.2)	43 (35.8)		78 (34.7)	
>34	74 (28.8)	40 (33.3)		75 (33.3)	
**Maternal Race**					
White	230 (89.5)	97 (80.8)		172 (76.4)	
Other	15 (5.8)	13 (10.8)	0.069	21 (9.3)	<0.001
Missing	12 (4.7)	10 (8.3)		32 (14.2)	
**Maternal Ethnicity**					
Hispanic	12 (4.7)	10 (8.3)	0.239	26 (11.6)	0.009
**Maternal Education, years**					
≤15	103 (40.1)	38 (31.7)	0.145	67 (29.8)	0023
>15	154 (59.9)	82 (68.3)		158 (70.2)	
**Family Socioeconomic Status**^**b**^					
Low (4 to 5)	22 (8.6)	8 (6.7)	0.654	17 (7.7)	0.827
High (1 to 3)	233 (91.4)	112 (93.3)		205 (92.3)	
**Country of Residence**					
U.S.	236 (91.8)	109 (90.8)	0.807	207 (92.0)	0.928
Canada	20 (7.8)	11 (9.2)		16 (7.1)	
**Region in U.S.**					
Northeast	36 (15.7)	26 (24.1)		27 (13.0)	<0.001
Midwest	69 (30.1)	20 (18.5)	0.002	32 (15.5)	
South	78 (34.1)	25 (23.1)		41 (19.8)	
West	46 (20.1)	37 (34.3)		107 (51.7)	
**Referral Source**					
Provider or Pharma	143 (55.9)	49 (40.8)		5 (2.2)	
Internet, Patient Support	95 (37.1)	38 (31.7)	<0.001	12 (5.3)	<0.001
OTIS Member Service	18 (7.0)	33 (27.5)		208 (92.4)	
**Year of Enrollment**					
2004–2006	30 (11.7)	47 (39.2)		26 (11.6)	
2007–2009	99 (38.5)	17 (14.2)	<0.001	87 (38.7)	0.999
2010–2016	128 (49.8)	56 (46.7)		112 (49.8)	
**Gestational Age at Enrollment in Study–mean (standard deviation)**	10.1 (4.2)	11.6 (4.3)	0.001	12.8 (4.3)	<0.001
Gravidity >1	149 (58.0)	81 (67.5)	0.098	138 (61.3)	0.512
Parity >0	119 (46.3)	64 (53.3)	0.245	113 (50.2)	0.443
Previous Spontaneous Abortion (Any)	60 (23.3)	36 (30.0)	0.210	54 (24.0)	0.951
Previous Elective Termination (Any)	21 (8.2)	12 (10.0)	0.680	19 (8.4)	0.681
**Pre-pregnancy Body Mass Index**^**e**^					
<24.9	157 (61.1)	83 (69.2)		148 (66.1)	
25–29.9	70 (27.2)	24 (20.0)	0.272	47 (21.0)	0.279
≥30	30 (11.7)	13 (10.8)		29 (12.9)	
Alcohol (Any)	101 (39.3)	55 (45.8)	0.277	98 (43.6)	0.393
Tobacco (Any)	33 (12.8)	12 (10.0)	0.534	6 (2.7)	<0.001
Folic Acid Containing Supplements—started before conception	168 (65.4)	85 (70.8)	0.350	150 (66.7)	0.839
Intended Pregnancy	173 (67.3)	77 (64.2)	0.627	171 (76.0)	0.045
Previous Child with a Birth Defect	9 (3.5)	11 (9.2)	0.041	7 (3.1)	1.000
Previous Preterm Delivery	24 (9.3)	12 (10.0)	0.988	18 (8.0)	0.720
**Prenatal Diagnosis Prior to Enrollment**					
Ultrasound Level II	10 (3.9)	4 (3.3)	1.000	13 (5.8)	0.450
Chorionic Villus Sampling	3 (1.2)	1 (0.8)	1.000	2 (0.9)	1.000
Amniocentesis	1 (0.4)	1 (0.8)	0.536	3 (1.3)	0.344
In Vitro Fertilization with this Pregnancy	11 (4.3)	5 (4.2)	1.000	8 (3.6)	0.862
**Comorbidities**					
Thyroid Disease	21 (8.2)	13 (10.8)	0.517	17 (7.6)	0.936
Asthma	24 (9.3)	8 (6.7)	0.504	14 (6.2)	0.273
Other Autoimmune Disease(s)	35 (13.6)	16 (13.3)	1.000	1 (0.4)	<0.001
Pre-gestational Hypertension	14 (5.4)	3 (2.5)	0.309	1 (0.4)	0.004
Psychiatric Condition	47 (18.3)	24 (20.0)	0.799	18 (8.0)	0.002
Any Infection in Pregnancy	175 (68.1)	92 (76.7)	0.125	174 (77.3)	0.036
Years Since Diagnosis of Rheumatoid Arthritis or Crohn’s Disease–mean (standard deviation)	8.4 (6.4)	8.3 (7.4)	0.910		
Exposure to a Known Human Teratogen	13 (5.1)	4 (3.3)	0.627	1 (0.4)	0.006
Systemic Steroid Use (Any)	91 (35.4)	55 (45.8)	0.068	1 (0.4)	<0.001
**Total Number of Weeks Exposed to Any Systemic Steroid Across Gestation**					
>1 to 4 weeks	23 (9.1)	10 (8.4)			
4.1 to 12 weeks	25 (9.9)	10 (8.5)	0.030		
>12 weeks	39 (15.4)	34 (28.8)			
Exposure to another anti-TNF-α medication	0	18 (15.0)			
**Exposure to other immunosuppressant medication (any)**	27 (10.5)	12 (10.0)	1.000		
Azathioprine	19 (7.4)	6 (5.0)	0.517		
6-mercaptopurine	8 (3.1)	6 (5.0)	0.388		
Disease Severity: HAQ-DI at Enrollment–mean (standard deviation)	0.4 (0.5)	0.6 (0.7)	0.036		
Disease Severity: Pain at Enrollment–mean (standard deviation)^j^	26.5 (26.7)	31.8 (28.9)	0.213		
Disease Severity: Global Impact at Enrollment–mean (standard deviation)	24.5 (26.1)	28.1 (28.3)	0.394		
Disease Severity: HAQ-DI in 3^rd^ Trimester–mean (standard deviation)	0.4 (0.5)	0.5 (0.5)	0.305		
Disease Severity: Pain In 3^rd^ Trimester–mean (standard deviation)	23.1 (26.6)	23.4 (24.6)	0.951		
Disease Severity: Global Impact in 3^rd^ Trimester–mean (standard deviation)	19.5 (24.3)	22.0 (23.2)	0.609		
Disease Severity: SIBDQ at Enrollment–mean (standard deviation)	5.5 (0.8)	5.6 (0.8)	0.497		
Disease Severity: SIBDQ in 3^rd^ Trimester–mean (standard deviation)	5.8 (0.9)	6.0 (0.5)	0.156		
Dose of Adalimumab per Treated Week–mg/week–mean (standard deviation)	32.4 (90.3)				
**Number of Weeks any Dose of Adalimumab Received in Pregnancy**					
>0–4	31 (12.7)				
4.1–6	9 (3.5)				
6.1–12	19 (7.4)				
>12	186 (75.9)				
**Gestational Timing any Dose of Adalimumab Received**					
1^st^ Trimester Only	55 (22.4)				
1^st^ and 2^nd^ Trimester	27 (10.5)				
1^st^ and 3^rd^ Trimester	3 (1.2)				
1^st^, 2^nd^ and 3^rd^ Trimester	160 (65.3)				

^a^Two-sample t-test for continuous variables; chi-squared test for categorical variables or Fisher’s Exact Test where expected cell number <5

^b^Socioeconomic status categorized 1–5 using Hollingshead method based on maternal and paternal education and occupation

Women in the adalimumab-exposed cohort compared to the disease comparison group were more likely to enroll earlier in gestation, to have been referred by their health care provider or the study sponsor. Women in the adalimumab-exposed cohort were less likely to have had a previous child with a birth defect, and reported fewer weeks of treatment with systemic corticosteroids (Table 1). Women in the adalimumab-exposed cohort with rheumatoid arthritis on average had lower scores on the HAQ-DI at enrollment than the disease comparison group, but other disease severity/symptom control measures did not differ between groups (Table 1).

A total of 25/602 (4.1%) of pregnancies resulted in live born twins. There were no stillbirths reported in any cohort; one elective termination was reported in the adalimumab-exposed group (Table 2). Among pregnancies ending in live birth, 22 of the 221 (10.0%) who had first-trimester adalimumab exposure were classified as having a major structural birth defect compared to 8/106 (7.5%) in the diseased unexposed group (adjusted OR 1.10, 95% CI 0.45 to 2.73) (Table 3). In the healthy comparison group, 12/198 (6.1%) of liveborn infants were classified with major structural birth defects (adjusted OR 1.43, 95% CI 0.33 to 6.27).

**Table 2:** Pregnancy outcomes of women exposed to adalimumab compared to women not exposed, 2004–2016.

Outcome	AdalimumabExposed N = 257 n/N (%)	Diseased Unexposed N = 120 n/N (%)	HealthyUnexposed N = 225 n/N (%)
**Live birth**	221/257 (86.0)	106/120 (88.3)	198/225 (88.0)
Singleton	208/221 (94.1)	100/106 (94.3)	192/198 (97.0)
Male Sex	112/207 (53.8)	51/100 (51.0)	110/192 (57.3)
Twin	13/221 (5.9)	6/106 (5.7)	6/198 (3.0)
Delivery by Caesarian Section	107/220 (48.4)	44/106 (41.5)	57/198 (28.8)
Stillbirth	0	0	0
Elective Termination	1/257 (0.4)	0	0
Lost-to-Follow-up	10/257 (3.9)	9/120 (7.5)	23/225 (10.2)

**Table 3:** Birth prevalence of major structural birth defects in pregnancies exposed to adalimumab compared to unexposed pregnancies, 2004–2016.

**Major Birth Defects**	**Adalimumab Exposed** **n/N (%)**	**Diseased Unexposed** **n/N (%)**	**Unadjusted RR** **(95% CI)**	**Adjusted OR** **(95% CI)**	**Healthy Unexposed** **n/N (%)**	**Unadjusted RR** **(95% CI)**	**Adjusted OR** **(95% CI)**
In Pregnancies Ending in at Least 1 Live Born Infant	22/221 (10.0)	8/106 (7.5)	1.32 (0.61 to 2.86)	1.10 (0.45 to 2.73)^a^	12/198 (6.1)	1.64 (0.83 to 3.23)	1.43 (0.33 to 6.27)^c^
Among All Pregnancies Excluding Lost-to-Follow-up	25/247 (10.1)	9/111 (8.1)	1.25 (0.60 to 2.59)	0.84 (0.34 to 2.05)^b^	12/202 (5.9)	1.70 (0.88 to 3.31)	1.33 (0.32 to 5.53)^d^
**Minor Birth Defects**							
Infants Examined with 3 or more of any minor structural birth defects	38/173 (22.0)	26/89 (29.2)	0.75 (0.49 to 1.16)	0.59 (0.31 to 1.12)^e^	39/154 (25.3)	0.87 (0.58 to 1.29)	0.49 (0.26 to 0.93)^f^
Infants with specific cluster of minor structural defects	4/173 (2.3%)	0/89			0/154		
**Among Women with Rheumatoid Arthritis**							
**Major Birth Defects**	**Adalimumab Exposed** **n/N (%)**	**Diseased Unexposed Unexposed** **n/N (%)**	**Unadjusted RR** **(95% CI)**		**Healthy Unexposed** **n/N (%)**	**Unadjusted RR** **(95% CI)**	
In Pregnancies Ending in at Least 1 Live Born Infant	6/69 (8.7)	5/74 (6.8)	1.29 (0.41 to 4.03)		12/198 (6.1)	1.43 (0.56 to 3.68)	
Among All Pregnancies Excluding Lost-to-Follow-up	6/76 (7.9)	6/77 (7.8)	1.01 (0.34 to 3.00)		12/202 (5.9)	1.33 (0.52 to 3.42)	
**Among Women with Crohn’s Disease**							
**Major Birth Defects**	**Adalimumab Exposed** **n/N (%)**	**Diseased Unexposed** **n/N (%)**	**Unadjusted RR** **(95% CI)**		**Healthy Unexposed** **n/N (%)**	**Unadjusted RR** **(95% CI)**	
In Pregnancies Ending in at Least 1 Live Born Infant	16/152 (10.5)	3/32 (9.4)	1.12^g^ (0.38 to 4.45)		12/198 (6.1)	1.74 (0.85 to 3.56)	
Among All Pregnancies Excluding Lost-to-Follow-up	19/171 (11.1)	3/34 (8.8)	1.26^h^ (0.44 to 4.07)		12/202 (5.9)	1.87 (0.93 to 3.74)	

^a^ Directly adjusted for year of enrollment (categorical)

^b^Adjusted for propensity score comprised of gestational age at enrollment and referral source

^c^ Adjusted for propensity score comprised of gestational age at enrollment and referral source

^d^Adjusted for propensity score comprised of gestational age at enrollment and referral source

^e^Directly adjusted for year of enrollment (categorical)

^f^Adjusted for propensity score comprised of psychiatric conditions and referral source

^g^Unadjusted odds ratio from logistic regression 1.31 (95% CI 0.38 to 4.52)

^h^Unadjusted odds ratio from logistic regression 1.14 (95% CI 0.31 to 4.16)

Among all pregnancies excluding those lost-to-follow-up, there were 25/247 (10.1%) first-trimester adalimumab-exposed pregnancies that resulted in at least one fetus/infant classified as having a major birth defect. In the diseased unexposed group, 9/111 (8.1%) resulted in at least one fetus/infant with a major birth defect (adjusted OR 0.84, 95% CI 0.34 to 2.05). In the healthy unexposed group, 12/202 (5.9%) resulted in at least one fetus/infant with a major birth defect (adjusted OR 1.33 95% CI 0.32 to 5.53 (Table 3). Following review by the investigators and consultation with the Scientific Advisory Board, there was no specific pattern of major structural defects identified in the adalimumab-exposed cohort (S3 Table).

Numbers of events in the two specific maternal disease strata were too limited to allow for statistical adjustment for confounders. However, the unadjusted RR for major birth defects among livebirths in adalimumab-exposed women within the rheumatoid arthritis stratum compared to unexposed women with rheumatoid arthritis was 1.29 (95% CI 0.41 to 4.03). In the Crohn’s Disease stratum, the unadjusted RR for major birth defects among live births in adalimumab-exposed compared to disease comparison pregnancies was 1.12 (95% CI 0.38 to 4.45) (Table 3).

Sensitivity analyses using the five alternative methods for adjusted analysis or restriction of the sample produced similar results (data not shown). A total of 416/543 (76.6%) of live born infants (including twins) received the study-specific dsymorphology examination. Reasons for non-participation in the physical examination were difficulty in scheduling the home visit or inability to contact the mother to schedule the visit. There were no significant differences between groups in the incidence of infants with three or more minor anomalies. Two sets of two infants in the adalimumab-exposed group had the same three minor structural defects. One set of two children each had an unruly hair pattern, bilateral epicanthal folds (folds of skin on inner corner of eye), and short palpebral fissures (smaller diameter of the eye opening). The other two infants with a cluster of the same three minor defects each had a prominent nasal bridge, a sacral dimple, and bilateral incurving of the 5^th^ fingers. One of these four children also had multiple major structural birth defects including asymmetry of the brain ventricles, bicuspid aortic valve, and congenital chordee. Neither of the same two clusters were identified in the comparison groups (Table 3).

The adjusted HR for spontaneous abortion in the adalimumab-exposed cohort compared to the diseased unexposed cohort was 2.22 (95% CI 0.67 to 7.29). The adjusted HR was somewhat higher in comparison to the healthy cohort, but with wide confidence intervals (adjusted HR 4.27, 95% CI 0.96 to 18.95). The adjusted HR for preterm birth was 0.82 (95% CI 0.66 to 7.20) in adalimumab-exposed pregnancies compared to the diseased unexposed group. However, in comparison to the healthy group, the adjusted HR for preterm delivery was elevated (2.59, 95% CI 1.22 to 5.50) (Table 4).

**Table 4:** Estimated probabilities of spontaneous abortion and preterm delivery in pregnant women exposed to adalimumab compared to women not exposed, 2004–2016.

**Outcome**	**Adalimumab Exposed** **N = 257**	**Diseased** **Unexposed** **N = 118**	**Unadjusted HR** **(95% CI)**	**Adjusted HR** **(95% CI**	**Healthy Unexposed** **N = 222**	**Unadjusted HR** **(95% CI)**	**Adjusted HR** **(95% CI)**
Number of Spontaneous Abortions	25	5			4		
Left Truncation Accounted Rate and HR (95% CI)	16.9% (11.5% to 24.3%)	11.1% (4.5% to 25.8%)	1.76 (0.67 to 4.61)	2.22 (0.67 to 7.29)^a^	5.2% (1.8% to 14.9%)	3.38 (1.17 to 9.76)	4.27 (0.96 to 18.95)^b^
**Outcome**^**c**^	**Adalimumab Exposed** **N = 213**	**Diseased Unexposed** **N = 100**	**Unadjusted HR** **(95% CI)**	**Adjusted HR** **(95% CI**	**Healthy Unexposed** **N = 203**	**Unadjusted HR** **(95% CI)**	**Adjusted HR** **(95% CI)**
Number of Preterm Deliveries	24	14			10		
Preterm Delivery Rate and HR (95% CI^)^	11.5% (7.9% to 16.7%)	14.0% (8.5% to 22.5%)	0.82 (0.43 to 1.59)	0.82 (0.66 to 7.20)^d^	5.1% (2.8% to 9.4%)	2.29 (1.09 to 4.78)	2.59 (1.22 to 5.50)^e^

^a^ Adjusted for propensity score comprised of years since diagnosis of primary disease (rheumatoid arthritis or Crohn’s disease), maternal age (categorical), and total number of weeks exposed to systemic steroids prior to 20 weeks

^b^ Adjusted for propensity score comprised of maternal age (categorical), tobacco use and infections prior to 20 weeks’ gestation, and referral source

^c^Analyses of preterm delivery restricted to pregnancies ending in singleton live births

^d^ Adjusted for propensity score comprise of total number of weeks exposed to systemic steroids prior to 37 weeks’ gestation and year of enrollment (categorical)

^e^ Directly adjusted for maternal race

Infants ≤10^th^ centile at birth or at approximately one year of age on weight, length and head circumference did not differ significantly in any between-group comparisons (Table 5).

**Table 5:** Pre- and postnatal growth deficiency in singleton infants born to women exposed to adalimumab compared to unexposed, 2004 to 2016.

≤10^th^ centile for Gestational Age At Birth	Adalimumab-Exposed n/N (%)	Diseased Unexposed n/N (%)	Unadjusted RR (95% CI)	Adjusted OR (95% CI)	Health Unexposed n/N (%)	Unadjusted RR (95% CI)	Adjusted OR (95% CI)
Weight	17/206 (8.3)	11/100 (11.0)	0.75 (0.37 to 1.54)		15/192 (7.8)	1.06 (0.54 to 2.06)	1.07 (0.52 to 2.21)^b^
Length	13/205 (6.3)	6/99 (6.1)	1.05 (0.41 to 2.67)		8/190 (4.2)	1.51 (0.64 to 3.55)	
Head Circumference	29/182 (15.9)	10/90 (11.1)	1.43 (0.73 to 2.81)	1.46 (0.67 to3.15)^a^	17/165 (10.3)	1.55 (0.88 to 2.71)	1.59 (0.84to 3.03)^c^
≤**10**^**th**^ **Centile at 1 Year of Age**							
Weight	30/168 (17.9)	11/78 (14.1)	1.27 (0.67 to 2.39)	1.17 (0.54 to 2.51)^d^	32/144 (22.2)	0.80 (0.51 to 1.25)	0.90 (028 to 2.85)^e^
Length	13/167 (7.8)	6/77 (7.8)	1.00 (0.39 to 2.53)		9/143 (6.3)	1.24 (0.54 to 2.81)	
Head Circumference	9/161 (5.6)	2/75 (2.7)	2.10 (0.51 to 12.62)		0/138 (0)		

^a^ Adjusted for propensity score comprised of primary disease (rheumatoid arthritis or Crohn’s disease), total number of weeks exposed to systemic oral corticosteroid in pregnancy, year of enrollment (categorical), duration of adalimumab exposure (none, quite before third trimester, third trimester), and referral source

^b^ Adjusted for propensity score comprised of maternal race, duration of adalimumab exposure (none, quite before third trimester, third trimester), and referral source

^c^ Adjusted for propensity score comprised of duration of adalimumab exposure (none, quite before third trimester, third trimester), and referral source

^d^ Directly adjusted for average dose of systemic oral corticosteroid in pregnancy

^e^ Adjusted for propensity score comprised of gestational age at enrollment and referral source

Serious or opportunistic infections in the first year of life were infrequent, occurring in 20/543 (3.7%) of live born infants. There were no significant differences between the adalimumab-exposed group compared to the diseased unexposed group (unadjusted RR 0.97, 95% CI 0.34 to 2.77) or to the healthy comparison group (unadjusted RR 1.77, 95% CI 0.62 to 5.05). Unadjusted RRs were similar when restricting the comparisons to those in the adalimumab-exposed group to those with third-trimester use of the drug (data not shown). No malignancies were reported in any infant up to one year of age (Table 6).

**Table 6:** Serious or opportunistic infections and malignancies up to one year of age in infants born to adalimumab compared to women not exposed, 2004 to 2016.

	Adalimumab-Exposed n/N (%)	Diseased Unexposed n/N (%)	Unadjusted RR^a^ (95% CI)	Non-Diseased Unexposed n/N (%)	Unadjusted RR^b^ (95% CI)
Serious or Opportunistic Infections in Infants	10/229 (4.4)	5/111 (4.5)	0.97 (0.34 to 2.77)	5/203 (2.5)	1.77 (0.62 to 5.05)
Malignancies in Infants	0	0		0	

## Discussion

In this prospective U.S. and Canada-wide cohort study, in comparing adalimumab-exposed to disease-matched pregnancies, the overall risk for major structural birth defects was not increased among live births (adjusted OR 1.10, 95% CI 0.45 to 2.73). Furthermore, among the major structural birth defects identified in the adalimumab-exposed group, there was no evidence of a consistent pattern. There was evidence of an approximate doubling of risk for preterm delivery in adalimumab-exposed but only in comparison to the healthy unexposed women.

Previous publications on the risk of major structural birth defects following adalimumab exposure in pregnancy have been largely limited to small case series which are difficult to interpret. While Weber-Schoendorfer et al (2015) noted a borderline two-fold elevated risk for any major structural birth defect with any anti-TNF-α exposure, the authors did not find a differentially elevated risk or a specific pattern of defects in the subset of 177 women exposed to adalimumab. Furthermore, in that study, the comparison group was not matched on disease and therefore could not address the contribution of the maternal underlying condition [2]. In contrast, Broms et al (2016) compared 161 adalimumab-exposed pregnancies to women with chronic inflammatory disease but no TNF inhibitor treatment and did not find a significantly elevated risk (adjusted OR 1.22, 95% CI 0.58 to 2.27) [3].

The birth prevalence of major birth defects in live births ranged from 6.1% in the healthy comparison cohort to 10.0% in the adalimumab-exposed cohort, which is higher than the 3–5% general population rates typically reported. It is possible that erring on the side of caution in careful abstraction of medical records over the first year of life, as is typical in pregnancy registries, led to identification of more defects. In support of this, the birth prevalence of major defects in another similar sized pregnancy registry for an asthma biologic, omalizumab, was 8.1% in the exposed and 8.9% in the comparison group [17]. In addition, relatively common anomalies, such as ventricular septal defects, were counted as malformations whether or not they were known to have subsequently resolved. However, we uniformly applied the Centers for Disease Control Metropolitan Atlanta criteria for classification of major birth defects across all three cohorts, leading to internal consistency.

The overall prevalence of three or more of any minor anomalies in the subset of infants who received the dysmorphology examination was reduced in the adalimumab-exposed cohort compared to the healthy cohort. However, two clusters of two infants with the same three minor defects were identified among the adalimumab-exposed who had three or more minor anomalies, one of which had associated major structural birth defects. The minor defects evaluated in the study examination in and of themselves have no clinical or cosmetic importance and could be familial. The purpose of this level of evaluation was to identify potential patterns of major and minor defects that may represent a teratogenic effect. While no child with either of these clusters was identified in the comparison groups, the finding of these minor anomalies in the adalimumab-exposed group could be due to chance.

In the current study, we also did not find significantly increased risks for spontaneous abortion, preterm delivery, pre- or postnatal growth restriction, serious or opportunistic infections, or malignancies when comparing adalimumab-exposed to disease comparison pregnancies/infants.

Similarly, there were no differences in comparisons for these same outcomes between the adalimumab-exposed group and the healthy cohort, except for an approximate doubling of risk for preterm delivery (adjusted HR 2.59, 95% CI 1.22 to 5.50). This isolated finding is consistent with several previous studies in women with rheumatoid arthritis or Crohn’s Disease, suggesting that the diseases themselves contribute to the risk of early delivery.

Using a Norwegian register, Wallenius et al (2013) noted an increased risk of preterm delivery among first pregnancies in 1,496 women with rheumatoid arthritis compared to 625,642 deliveries from the general population (adjusted OR 1.5, 95% CI 1.1 to 2.0) [18]. Similarly, Norgaard et al (2010) examined 1,199 pregnancies in women with rheumatoid arthritis compared to 870,380 women without in Denmark and Sweden and found increased risks for moderate preterm delivery (32 and 36 weeks’ gestation) (adjusted OR 1.44, 95% CI 1.14 to 1.82) and very preterm delivery (<32 weeks’ gestation) (adjusted OR 1.55, 95% CI 0.97 to 2.47) [19].

An analysis of Danish Birth Cohort data found that among 644 women with Inflammatory Bowel Disease (including 266 with Crohn’s Disease), there was an approximate two-fold risk for preterm delivery compared to 83,795 with no bowel disease [20]. Similarly, Stephansson et al (2010) examined data from 2,377 pregnant women with Crohn’s Disease and 869,202 pregnant women without from Danish and Swedish medical birth registries and found increased risks for both moderate and very preterm delivery (OR 1.76; 95% CI, 1.51 to 2.05; and OR 1.86; 95% CI, 1.38 to 2.52, respectively) [21].

We found no evidence of an increased risk for small for gestational age infants with adalimumab exposure or among women with the underlying diseases we studied. Although women enrolled in the study were of higher socioeconomic status and predominately white, rates of small for gestational age infants on birth weight were within the range of the expected population rate of 10% in all cohorts. In contrast, Norgaard et al (2010) noted a modest increased risk of small for gestational age on birth weight (OR 1.56, 95% CI 1.20 to 2.01) among infants born to women with rheumatoid arthritis, but the overall incidence of smaller birth weight in this population-based sample was low (3.8% to 5.9%) [19].

Although we found no significant increases in spontaneous abortion in any comparison, the point estimates for the HRs were well above 1 with very wide confidence intervals. However, as women enrolled on average at the end of the first trimester, we were not able to evaluate spontaneous abortion in the early weeks of gestation when the risk is highest. Further study of this outcome is needed.

It is generally thought that little anti-TNF medication crosses the placenta in the first trimester. However, there is direct and indirect evidence of placental transfer of anti-TNF-α medications in the third trimester via measurable cord blood levels even weeks after maternal discontinuation of treatment [1]. One case report suggested that adalimumab was detectable in the cord serum of an infant delivered at 37.5 weeks gestation despite the fact that the mother discontinued treatment in gestational week 16 [22].

We did not measure cord blood or infant levels of adalimumab or infant immune function in this study. However, we did examine anytime exposure and exposure near the time of delivery to adalimumab with respect to risk of serious or opportunistic infections in the first year of life, and did not find support for this theoretical concern. This finding is consistent with a U.S. claims database analysis for anti-TNF-α medications in general and adalimumab specifically [23]. In that study, among 108 women with rheumatoid arthritis who had adalimumab-exposed offspring, 1.9% were documented as having a serious infection requiring hospitalization. This was similar to the 2.0% rate of serious infections in infants of women with rheumatoid arthritis but no exposure to an anti-TNF-α medication in pregnancy who were ascertained from the same claims data sources.

There are several limitations to this study. First, the sample sizes of pregnancy registries, including this one, are typically insufficient to rule out elevated risks for most specific individual major birth defects. However, specific patterns of major structural birth defects are characteristic of known human teratogens. Therefore, it is reassuring that no such pattern was identified in this study. Secondly, we relied on volunteers, most of whom were white and of higher socioeconomic status, which could introduce bias and affect generalizability of the study results. However, pregnant women were enrolled in all three cohorts before the known outcome of pregnancy and similar data collection was completed for the comparison groups thereby supporting internal validity. We did not follow infants beyond one year of age and therefore were unable to assess longer term risk for malignancies or obtain data on child neurodevelopment. As with all studies of prenatal exposures, data on longer term outcomes including neurodevelopment would be valuable.

In summary, in this study, we found no evidence of an increased risk of major structural birth defects as well as a wide range of other pregnancy outcomes attributable to prenatal exposure to adalimumab. In addition, for women being treated for rheumatoid arthritis or Crohn’s Disease, this study confirms previous findings regarding elevated risks for preterm delivery that are associated with the maternal disease.

## Supporting information

S1 Fig Counts of women exposed to adalimumab in each week of gestational age. (DOCX)

S1 Table Checklist for minor structural defects. (DOCX)

S2 Table Checklist for serious or opportunistic infections. (DOCX)

S3 Table Listing of specific major structural birth defects. (DOCX)

S4 Table Details on missing covariates. (DOCX)
