# Supplementary figures and images for: Birth outcomes in women who have taken adalimumab in pregnancy: A prospective cohort study
Source: PLoS One. 2019 Oct 18;14(10):e0223603. doi: 10.1371/journal.pone.0223603 (PMC6799916; doi:10.1371/journal.pone.0223603)

**Supplemental Figure e1. Counts of Women Exposed to Adalimumab in Each Week of Gestational Age**

**
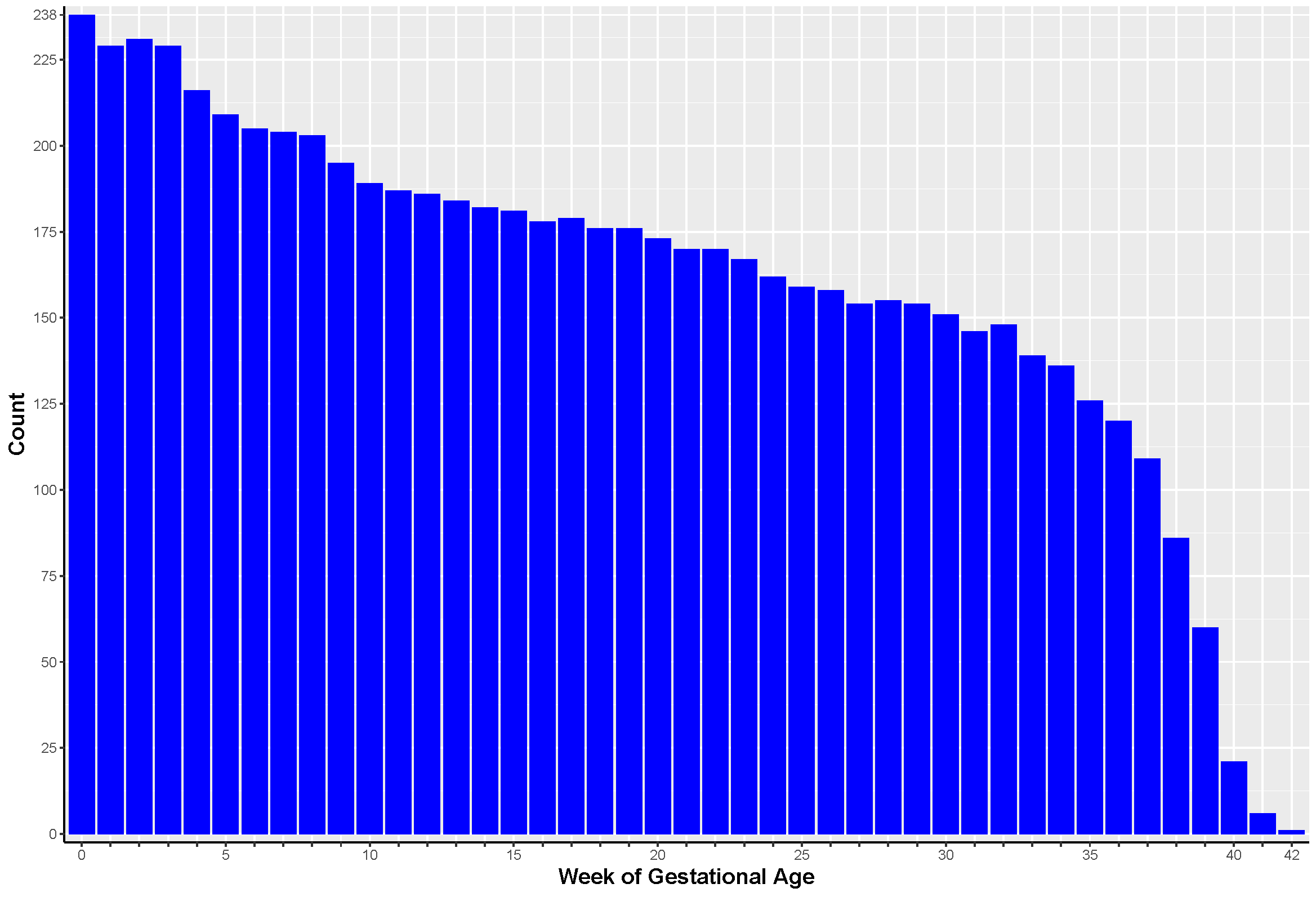
**

Supplement: S1 Fig — (DOCX) [file pone.0223603.s001.docx]
