# Supplementary material for: Birth outcomes in women who have taken adalimumab in pregnancy: A prospective cohort study
Source: PLoS One. 2019 Oct 18;14(10):e0223603. doi: 10.1371/journal.pone.0223603 (PMC6799916; doi:10.1371/journal.pone.0223603)
Supplement: S1 Table — (DOCX) [file pone.0223603.s002.docx]

**Supplemental Table e1. Checklist for Minor Structural Defects**

| **Calvarium** |
| --- |
| Bifrontal Diameter Narrow |
| Frontal Bossing |
| Metopic Ridge |
| Metopic Suture Open |
| Third Fontanel |
| Large Anterior Fontanel |
| Occiput Prominent |
| Occiput Flat |
| Hair Whorl Double |
| Hair Whorl Triple |
| Hair Whorl Absent |
| Hair Whorl Midline |
| Hair Pattern Unruly |
| Frontal Upsweep |
| Widow’s Peak |
| Depigmentary Hair Changes |
| Scalp Defect |
| Plagiocephaly |
| Sutural Synostosis |
| Other |
| **Face** |
| Supraorbital Ridges |
| Prominent |
| Hypoplastic |
| Eyebrows |
| Synophrys |
| Medial Flare |
| Nasal Bridge |
| Flat |
| Prominent |
| Nostrils |
| Anteverted |
| Hypoplastic |
| Thickened |
| Philtrum |
| Smooth |
| Vermillion Thin |
| Cupid’s Bow |
| Facial Asymmetry |
| Maxillary Hypoplasia |
| Other |
| **Eyes** |
| Epicanthal Folds |
| Left |
| Right |
| Ptosis |
| Left |
| Right |
| Iris Defect |
| Eyelashes |
| Absent |
| Defect |
| Strabismus |
| Other |
| **Mouth** |
| Multiple Frenuli |
| Broad Alveolar Ridge |
| *CLEFT LIP* |
| Cleft Alveolar Ridge |
| *CLEFT PALATE* |
| Cleft Uvula |
| Micrognathia |
| Prognathia |
| Macroglossia |
| Microglossia |
| Prominent Lingual Frenuli |
| Other |
| **Ears** |
| Preauricular Pit |
| Left |
| Right |
| Preauricular Tag |
| Left |
| Right |
| Altered Shape / Position |
| Other |
| **Neck** |
| Webbed |
| Short |
| Broad |
| Low Post Hair Line |
| Hair Upsweep |
| Branchial Sinus |
| Torticollis |
| Other |
| **Chest and Abdomen** |
| Supernumerary Nipples |
| Left |
| Right |
| Xiphoid Bifid |
| Poland Sequence |
| Clavicles Absent |
| Pectus Excavatum |
| Pectus Carinatum |
| Diastasis Recti |
| Umbilical Hernia |
| Other |
| **Genitalia - Male** |
| Undescended Testes |
| Left |
| Right |
| Testes in Groin |
| Left |
| Right |
|  |
| Hydrocele: communicating |
| non-communicating |
| *HYPOSPADIAS*(circle): 1°, *2°, 3°* |
| Chordee |
| *EPISPADIAS* |
| Scotalization of Phallus |
| Scrotum Shawl |
| Scrotum Bifid |
| Absent Median Ridge |
| Micropenis |
| Other |
| **Genitalia - Female** |
| Labia Majora Hypoplastic |
| Labia Minora Hypoplastic |
| Clitorimegaly |
| Median Raphe Present |
| Other |
| **Back** |
| Sacral Dimple |
| Coccygeal Dimple |
| Cutaneous Marker Midline |
| Other |
| **Anus** |
| Anteriorly Placed |
| *IMPERFORATE* |
| Other |
| **Arms** |
| Inability to fully extend elbows |
| Cubitus Valgus |
| Pterygia |
| Radioulnar Synostosis |
| Shoulder Dimples |
| Other |
| **Hands** |
| Clinodactyly 5^th^ Finger |
| Left |
| Right |
| Thumb Hypoplasia |
| Left |
| Right |
| Fingers Tapered |
| Fingers Overlapping |
| Finger Tip Pads Prominent |
| Fingernails |
| Hypoplastic |
| Hyperconvex |
| Simian Crease |
| Left |
| Right |
| Aberrant Creases Other |
| Interphalangeal Creases Absent |
| Left PIP: 2_3_4_5_ |
| Left DIP: 2_3_4_5_ |
| Right PIP: 2_3_4_5_ |
| Right DIP: 2_3_4_5_ |
| IP Creases Extra |
| Fingers |
| Left |
| Right |
| Thenar Crease Absent |
| Left |
| Right |
| *POLYDACTYLY* |
| Syndactyly |
| Other |
| **Legs** |
| Genu Valgum |
| Patella Absent |
| Other |
| **Feet** |
| Space increase 1-2 toes |
| Left |
| Right |
| Syndactyly 2-3 |
| Left |
| Right |
| Toes Overlapping 5^th^ over 4th |
| Left |
| Right |
| Toes Overlapping – Other |
| Left |
| Right |
| Toenails Hypoplastic |
| Heels Prominent |
| Left |
| Right |
| Vertical Sole Crease |
| Left |
| Right |
| Metatarsus adductus |
| Left |
| Right |
| Calcaneovalgus |
| Left |
| Right |
| *POLYDACTYLY* |
| Other |
| **Skin** |
| Capillary Hemangioma (count only if > 1.5 cm) |
| Vascular Malformation |
| Glabella |
| Neck |
| Eye Lids |
| Crown |
| Other |
| Mongolian Spot |
| Sacrum |
| Back |
| Shoulders |
| Café-Au-Lait (count only if   1.5 cm or > 6 CAF spots) |
| Nevus Sebaceous |
| Hirsuitism |
| Dipigmentary Skin Changes |
| Other |
| **Neurologic** |
| Hypotonia |
| Hypertonia |
| Irritability |
| Other |
| **Joints** |
| Contracture |
| Laxity |
| Dislocation |
| Other |

|  | Measurements: |
| --- | --- |
| Length |  |
| Weight |  |
| OFC |  |
| Palpebral Fissure |  |
| Left |  |
| Right |  |
| Ears |  |
| Left |  |
| Right |  |
| Philtrum |  |
| Inner Canthal Distance |  |
