# Supplementary material for: Birth outcomes in women who have taken adalimumab in pregnancy: A prospective cohort study
Source: PLoS One. 2019 Oct 18;14(10):e0223603. doi: 10.1371/journal.pone.0223603 (PMC6799916; doi:10.1371/journal.pone.0223603)
Supplement: S2 Table — (DOCX) [file pone.0223603.s003.docx]

**Supplemental Table e2. Checklist for Serious or Opportunistic Infections**

| X-ray proven pneumonia (requiring antibiotic treatment and/or hospitalization) |
| --- |
| Neonatal sepsis |
| Meningitis |
| Osteomyelitis |
| Bacteremia Septic arthritis |
| Abscess (deep tissue) |
| Mycobacteria infections (incl. but not limited to tuberculosis) |
| Invasive fungal infection including histoplasmosis, coccidiomycosis, candidiasis, aspergillosis, blastomycosis |
| Pneumocystis jirovecii infection |
