# Supplementary material for: Birth outcomes in women who have taken adalimumab in pregnancy: A prospective cohort study
Source: PLoS One. 2019 Oct 18;14(10):e0223603. doi: 10.1371/journal.pone.0223603 (PMC6799916; doi:10.1371/journal.pone.0223603)
Supplement: S3 Table — (DOCX) [file pone.0223603.s004.docx]

**Supplemental Table e3. Listing of Specific Major Structural Birth Defects**

| Adalimumab Exposed – Rheumatoid Arthritis | 1. Undescended testes unilateral  2. Microcephaly  3. Microcephaly  4. Ventricular septal defect (resolved)  5. Dandy Walker Spectrum  6. Multiple hemangiomas |
| --- | --- |
| Adalimumab Exposed-  Crohn’s Disease | 7. Ventricular septal defect  8. Ventricular septal defect, peripheral pulmonic stenosis, atrial septal defect  9. Ventricular septal defect, hydrocephalus secondary to intraventricular hemorrhage, patent foramen ovale, patent ductus arteriosus, first degree hypospadias, bilateral hydronephrosis  10. Peripheral pulmonary arterial branch narrowing, microcephaly, patent ductus arteriosus, autosomal chromosomal deletion  11. Peripheral pulmonic stenosis  12. Hydronephrosis unilateral surgically repaired  13. Congenital hip dislocation surgically repaired, inguinal hernia  14. Pre-axial polydactyly  15. Flexion contraction of 3rd finger on one hand, surgically corrected  16. Hypospadias, degree not specified, surgically corrected  17. Ectopic thyroid, lingual thyroid gland  18. Larygomalacia with omega-shaped epiglottis and slit-like opening of the laryngeal introitus  19. Spina bifida cystica, hydrocephalus, Arnold-Chiari malformation  20. Asymmetry of ventricles, bicuspid aortic valve, congenital chordee, hypotonia  21. Ectopia cordis, kyphoscoliosis, caudal regression, bifid sternum in one member of twin pair spontaneously aborted  22. Trisomy 18  23. Congenital cataracts bilateral, ventricular septal defect, patent foramen ovale  24. Microcephaly  25. Mild cerebellar tonsillar hypoplasia, esotropia, nystagmus, blindness |
| Diseased Unexposed –  Rheumatoid Arthritis | 1. Large hemangioma, anteriorly placed anus  2. Talipes equinovarus bilateral, surgically corrected  3. Microcephaly  4. Chromosomal anomaly, not otherwise specified  5. Anterior polar cataract  6. Ventricular septal defect |
| Diseased Unexposed –  Crohn’s Disease | 7. Ventricular septal defect, patent foramen ovale, peripheral pulmonary artery stenosis  8. Ventricular septal defect  9. Fetal tachycardia, patent ductus arteriosus, paten foramen ovale, congenital ascites |
| Healthy Unexposed | 1.Ventricular septal defect, patent foramen ovale  2.Ventricular septal defect, patent foramen ovale, patent ductus arteriosus  3.Ventricular septal defect, patent foramen ovale  4. Congenital hypertrophic pyloric stenosis, surgically repaired  5. Post-axial polydactyly toes  6. Undescended testes unilateral, surgically repaired  7. Undescended testes unilateral, surgically repaired  8. Femoral bone deficiency unilateral  9. Congenital hypertrophic pyloric stenosis, surgically required  10. Congenital hypertrophic pyloric stenosis, surgically required  11. Trisomy 21  12. Trisomy 18 |
