# Supplementary material for: Birth outcomes in women who have taken adalimumab in pregnancy: A prospective cohort study
Source: PLoS One. 2019 Oct 18;14(10):e0223603. doi: 10.1371/journal.pone.0223603 (PMC6799916; doi:10.1371/journal.pone.0223603)
Supplement: S4 Table — (DOCX) [file pone.0223603.s005.docx]

**Supplemental Table e4 Details on Missing Covariates**

Race missing for 54 women, due to reclassification of Hispanic as ethnicity instead of race category.

SES missing for 2 women in Adalimumab-Exposed Group and 3 in the Healthy Unexposed Group.

Country of residence missing for 1 woman in the Adalimumab-Exposed Group and 2 in the Healthy Unexposed Group.

Region of the U.S. missing for 7 women in the Adalimumab-Exposed Group and 1 in the Diseased Unexposed Group

Pre-pregnancy body mass index missing for 1 woman in the Healthy Unexposed Group.

Years since first diagnosis of disease missing for 2 women in the Adalimumab-Exposed Group and 1 in the Diseased Unexposed Group.

Total number of weeks exposed to systemic steroids missing for 4 women in the Adalimumab-Exposed Group and 1 in the Diseased Unexposed Group.

Dose of oral systemic corticosteroids missing for 20 women in the Adalimumab-Exposed Group and 8 subjects in the Diseased Unexposed Group.

HAQ-DI missing at enrollment for 1 woman in the Adalimumab-Exposed Group; in the third trimester, missing for 26 women in the Adalimumab-Exposed Group and 1 woman in the Diseased Unexposed Group.

Pain score missing at enrollment for 1 woman in the Adalimumab-Exposed Group; in the third trimester, missing for 26 women in the Adalimumab-Exposed Group and 1 woman in the Diseased Unexposed Group.

Global impact core missing at enrollment for 1 woman in the Adalimumab-Exposed Group; in the third trimester, missing for 26 women in the Adalimumab-Exposed Group and 1 woman in the Diseased Unexposed Group.

SIBDQ scores missing at enrollment for 53 women in the Adalimumab-Exposed Group and 1 in the Diseased Unexposed Group; in the third trimester, missing for 68 women in the Adalimumab-Exposed Group and 14 women in the Diseased Unexposed Group.

Dose of adalimumab missing for 2 women in the Adalimumab-Exposed Group.

Duration of adalimumab exposure missing for 12 women in the Adalimumab-Exposed Group.
